# Analysis of Epidemiological and Molecular Characteristics of Bocavirus in Guangzhou

**DOI:** 10.3390/v18060686

**Published:** 2026-06-20

**Authors:** Yifan Pan, Pingting Zhu, Yiyun Chen, Jingjing Zhang, Yanhui Liu, Shuiping Hou, Anna Wang, Xinwei Wu, Pengzhe Qin, Lan Cao

**Affiliations:** 1 School of Public Health, Guangzhou Medical University, Guangzhou 511436, China; 15625853809@163.com; 2 Guangzhou Center for Disease Control and Prevention (Guangzhou Health Supervision Institute), Guangzhou 510440, China; ptzhu215@163.com (P.Z.); by_chenyiyun@gz.gov.cn (Y.C.); zhangjj75@mail3.sysu.edu.cn (J.Z.); gzcdc_liuyh@gz.gov.cn (Y.L.); gzcdc367@163.com (S.H.); anna1871@126.com (A.W.); tomwu@126.com (X.W.); petgyy@gmail.com (P.Q.); 3 School of Public Health, Southern Medical University, Guangzhou 510515, China

**Keywords:** human bocavirus, epidemiology, whole-genome sequencing, genetic recombination, antigenic variation

## Abstract

Objective: We aimed to elucidate the epidemiological characteristics and co-infection status of HBoV in Guangzhou and to investigate the potential recombination events and alterations in antigenic properties among circulating HBoV strains. Methods: Utilizing respiratory specimens collected from patients at sentinel surveillance hospitals in Guangzhou between August 2023 and December 2025, multiplex pathogen detection was performed. We describe the temporal and demographic distribution of HBoV in Guangzhou and determine its co-infection patterns. Subsequent sequence analysis focused on identifying potential recombination events and characterizing antigenic properties. Results: The epidemiological features of HBoV in Guangzhou exhibited a primary epidemic peak around the autumn season, followed closely by a secondary peak. HBoV infection was predominantly observed in children under three years of age. Co-infections with rhinovirus and parainfluenza virus were common. Whole-genome sequencing yielded 15 complete HBoV genome sequences. Recombination analysis and verification suggested potential recombination events in two of these sequences. A comparative analysis of the antigenic characteristics of one identified recombinant strain, GZ-2024-20891, against its putative parental strains and domestic prevalent strains revealed potential alterations in its antigenic characteristic. Conclusions: Bocavirus is highly prevalent among young children under 3 years of age, with a secondary peak following the main epidemic peaks around autumn in Guangzhou. Genetic recombination and potential antigenic alteration were detected in bocavirus.

## 1. Introduction

Respiratory infections are recognized as one of the leading causes of global morbidity and mortality, particularly in low- and middle-income countries, where they continue to constitute a significant source of fatality and disease burden. Despite recent advancements in prevention and control measures, the management of respiratory infections remains a major public health challenge that requires urgent resolution globally. Therefore, human bocavirus (HBoV), as a common respiratory pathogen, has attracted widespread attention regarding its epidemiological characteristics. HBoV was first discovered in 2005 by researchers through large-scale molecular screening of nasopharyngeal swab samples from children with acute respiratory infections [1]. Bocavirus is a non-enveloped, single-stranded DNA virus classified within the genus Bocaparvovirus of the family Parvoviridae and exhibits high genetic homology with bovine parvovirus and canine minute virus.

The genome of bocavirus is approximately 5.5 kb in length, featuring inverted terminal repeats (ITRs) with hairpin structures at both ends [2]. The central region contains three open reading frames (ORFs), through differential splicing of mRNA, which encode various non-structural proteins (NS), nucleoproteins (NP), and viral capsid proteins (VP) [3,4]. The NS and NP proteins, responsible for viral replication and transcriptional regulation, are relatively conserved in evolution. In contrast, the VP protein which constitutes the viral capsid exhibits greater variability [5,6]. Additionally, the viral genome encodes a small non-coding RNA named BocaSR, which has been demonstrated to promote the replication of the viral genome [7,8].

Phylogenetic analysis based on the VP1/2 gene sequences classifies HBoV into four genotypes (HBoV1-4) [9]. HBoV1 is the genotype most frequently associated with respiratory tract infections and has been linked to severe disease [10]. Epidemiological studies indicate that HBoV is detected worldwide, most commonly in children under three years of age, and often presents as a co-infection with other respiratory viruses [11,12,13].

To thoroughly understand the local epidemiology and molecular evolution of HBoV, this study systematically analyzed HBoV infections based on sentinel hospital surveillance in Guangzhou. We aimed to define the temporal and demographic patterns of HBoV, identify common co-infecting pathogens, and investigate molecular characteristics among circulating strains through whole-genome sequencing. These findings may provide a scientific basis for prevention and control of bocavirus infection.

## 2. Materials and Methods

### 2.1. Patients and Samples

From August 2023 to December 2025, nasopharyngeal swab samples were collected from 5316 patients with the criteria for influenza-like illness (ILI) and severe acute respiratory infection (SARI) from sentinel hospitals in Guangzhou. ILI cases were defined as outpatients or emergency department patients with acute onset of illness, body temperature ≥38 °C, and at least one of the following symptoms: cough or sore throat. SARI cases were defined as hospitalized patients with acute onset of illness at the time of admission or within 48 h after admission, a history of fever (body temperature ≥38 °C) at onset, presence of cough, and illness duration not exceeding 10 days. All samples were placed in collection tubes containing 3–4 mL of viral transport medium, stored at 2–8 °C, and transported within 48 h to the network laboratory of the Guangzhou Center for Disease Control and Prevention.

### 2.2. Nucleic Acid Extraction and Pathogen Screening

First, 200 µL of nasopharyngeal swab sample extract was transferred to a nucleic acid extraction plate (Jiangsu Bioperfectus Technologies, Taizhou, China) and processed according to the manufacturer’s instructions using a nucleic acid extraction instrument (Jiangsu Bioperfectus Technologies, China) for nucleic acid isolation. The NxTAG Respiratory Pathogen Panel (Luminex, Austin, TX, USA) was utilized to detect common respiratory pathogens in each specimen via bead-based multiplex RT-PCR, including bocavirus, influenza virus, respiratory syncytial virus, parainfluenza virus, rhinovirus, human metapneumovirus, adenovirus and SARS-CoV-2.

### 2.3. Complete Genome Amplifying and Sequencing

Complete genome amplification of bocavirus was performed using the PrimeScript™ II High Fidelity One Step RT-PCR Kit (TAKARA, Kyoto, Japan) with primers listed in reference [14] on the Bio-Rad PCR thermal cycler (Bio-Rad Laboratories, Hercules, CA, USA), with denaturation set at 98 °C for 10 s, annealing at 60 °C for 15 s, and extension at 68 °C for 30 s. Nucleic acid extracts from selected bocavirus-positive specimens were processed through sequential steps of genomic DNA Tagmentation, library amplification, library purification, library normalization, library pooling, and library dilution according to the methods described in the Nextera XT DNA Library Prep Kit Reference Guide, followed by loading 500 µL of the diluted library onto the Illumina MiniSeq sequencer (Illumina, San Diego, CA, USA) for complete genome sequencing. All bocavirus-positive specimens were subjected to whole-genome sequencing. Only those that generated complete genomes were included in this study.

### 2.4. Phylogenetic Tree Construction

Bocavirus reads obtained from sequencing were assembled using CLC Genomics Workbench V.22 (reference assembly: JN387083). Reference sequences of bocavirus genotypes, bovine parvovirus, and canine parvovirus were retrieved from NCBI GenBank and NCBI Virus databases. Multiple sequence alignment was performed using ClustalX V.2.1 program. Phylogenetic trees were constructed based on the bocavirus NS1, NP1, VP1/2 gene sequences and complete genome sequences in MEGA 11 software, using the neighbor-joining (NJ) method with the Maximum Composite Likelihood model and 1000 bootstrap replicates. All positions containing gaps and missing data were treated using pairwise deletion.

### 2.5. Recombination Detection

Potential recombination events in bocavirus sequences were detected using the Recombination Detection Program 4 (RDP4) V.4.101 [15], employing the following algorithms: RDP, GENECONV, BootScan, MaxChi, Chimaera, SiScan, and 3Seq. Recombining breakpoint identification was further validated using the BootScan algorithm in SimPlot software V.3.5.1 [16,17], with a window size of 1000 nt and a step size of 10 nt.

### 2.6. Linear Epitope Antigenicity Prediction

B cell linear epitopes of the bocavirus VP2 protein were predicted using ABCpred, BCpred, and Bepipred 3.0. For ABCpred, the prediction threshold was set at 0.51 and a peptide window length at 16 aa and an overlapping filter was enabled [18,19]. BCpred was configured with 75% specificity, 20 aa epitope length, and overlap filter activated [20]. Bepipred 3.0 was set at a threshold of 0.1512, selecting the top 20% of amino acid residues with higher confidence, with smoothing prediction enabled [21]. All parameters described above were used with their default settings.

CD8+ and CD4+ T cell epitopes of the VP2 protein were predicted using NetMHCIpan 4.1 and NetMHCIIpan 4.1 tools available through the Immune Epitope Database (IEDB) platform [22,23,24]. Both methods employed 27 HLA alleles covering 90% of the human population, with peptide lengths of 9 aa and 15 aa, respectively; the top 10% and top 25% percentile prediction results were selected for comparative analysis.

Predicted linear epitopes were subsequently uploaded to the Vaxijen 2.0 online server for antigenicity analysis [25], with a threshold of 0.85 for antigen prediction.

### 2.7. Conformational Epitope Modeling Prediction

The three-dimensional structural model of the bocavirus VP2 protein was constructed using the AlphaFold3 online server [26]. B cell conformational epitopes of the VP2 protein were predicted using the ElliPro tool available on IEDB [22,27], with minimum score and maximum distance set at 0.9 and 6 Å, respectively. Rendering of the VP2 protein structure was performed using PyMOL software V.3.6.1.

### 2.8. Statistical Analysis

Statistical analysis of the obtained data was performed using SPSS Statistics 25.0 software. A chi-square test or corrected chi-square test was employed to assess statistically significant differences between groups. For comparisons among k groups, chi-square partitioning was conducted with the significance level adjusted according to formula α’ = 2α/(k(k − 1)) to control for Type I error.

## 3. Results

### 3.1. Epidemiological Characteristics

**Figure 1:**
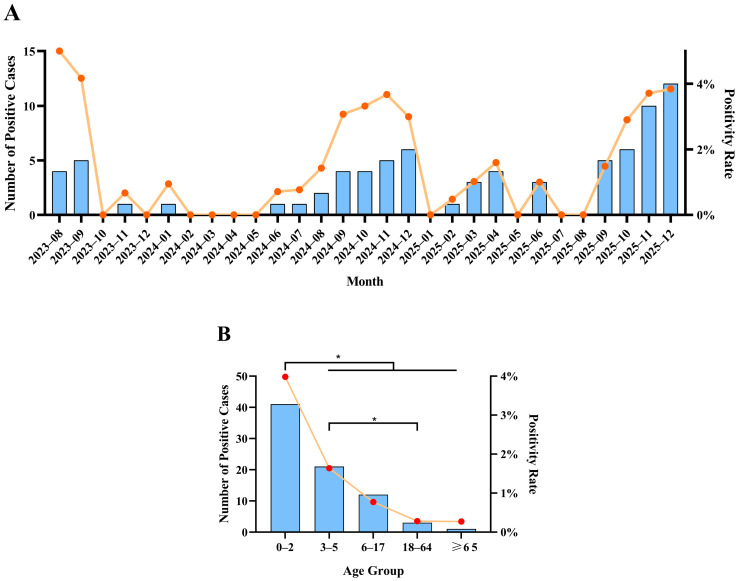
Seasonal distribution (**A**) and age distribution (**B**) of bocavirus in Guangzhou. Mark * indicates a comparison between age groups with *p* < 0.005.

A total of 5316 nasopharyngeal swab samples were collected from ILI and SARI cases at sentinel hospitals in Guangzhou from August 2023 to December 2025. The overall positivity rate for bocavirus was 1.47% (78/5316). As shown in Figure 1, bocavirus exhibited distinct seasonality, with the epidemic peak primarily occurring around autumn, followed closely by a secondary peak. Cochran–Armitage trend tests revealed significant linear trends in positivity rates across first peak, trough, and second peak in both the 2023 season (*χ*^2^ = 10.641, *p* = 0.001) and the 2024 season (*χ*^2^ = 6.902, *p* = 0.009), confirming the statistical significance of the second peaks in both epidemic cycles. The male-to-female ratio among bocavirus-positive cases was 43:35, with no statistically significant difference observed between gender (*χ*^2^ = 0.016, *p* = 0.900). Regarding age distribution, bocavirus-positive cases were mainly concentrated in children under three years of age, with a positivity rate of 3.98% (41/1030). The positivity rate in the under-three age group was significantly higher than in all other age groups (Table 1). Furthermore, the median age of bocavirus-positive cases was 2.25 years of age, and there was a trend of decreasing positivity rate with increasing age (Figure 1).

### 3.2. Co-Infection

**Figure 2:**
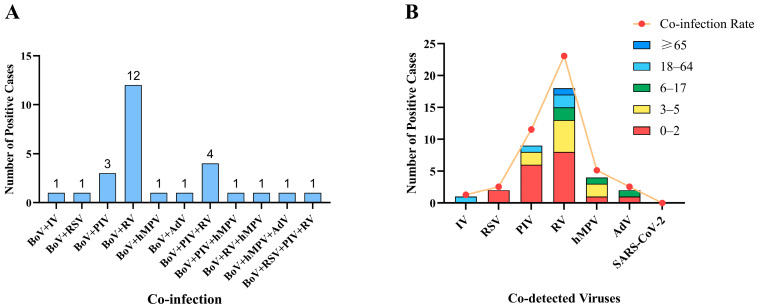
Detection of co-infection viruses with bocavirus (**A**) and age distribution of cases co-infected with bocavirus (**B**).

In addition to bocavirus, other common respiratory pathogens were detected in this study, including influenza virus, respiratory syncytial virus, parainfluenza virus, rhinovirus, human metapneumovirus, adenovirus and SARS-CoV-2 (Figure 2A). Among them, bocavirus and rhinovirus co-infection were the most common, with a co-infection rate of 23.08% (18/78). This was followed by parainfluenza virus, with a co-infection rate of 11.54% (9/78) (Table 2). Common co-infection events in both groups were more frequently observed in infants and young children under 3 years of age (Figure 2B). Among the cases of bocavirus co-infection, the main cases were dual infection, accounting for 70.37% (19/27), and the remaining cases were triple infections except for one quadruple infection, accounting for 25.93% (7/27).

### 3.3. Phylogenetic Analysis

**Figure 3:**
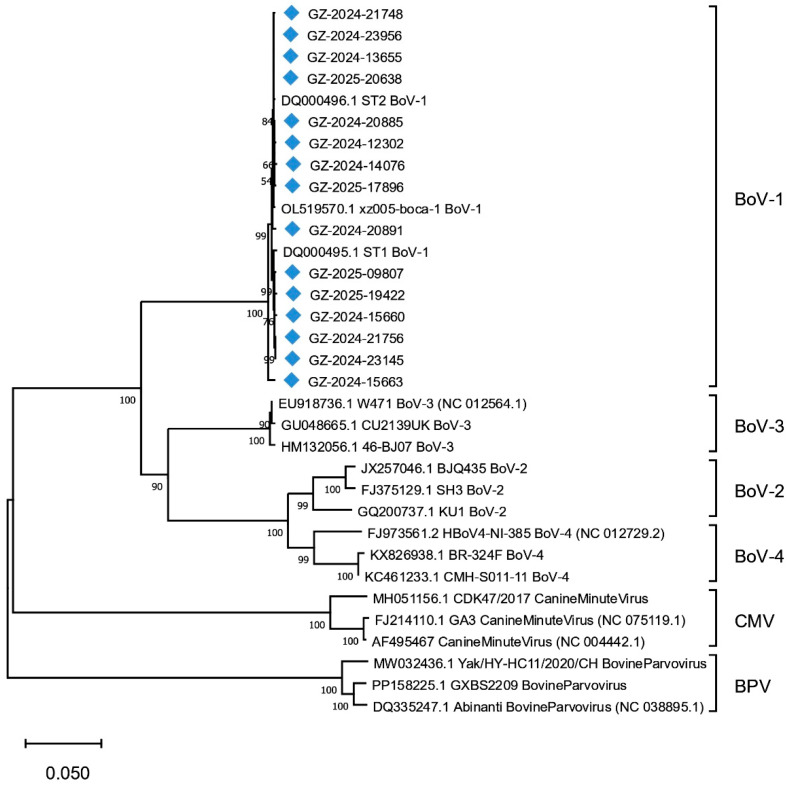
Phylogenetic analysis of human bocavirus whole genome. Mark ◆ denotes sequences obtained in this study.

In this study, 15 bocavirus sequences were obtained by whole-genome sequencing. The reference sequences employed for typing purposes encompass bocavirus type 1–4 and the sequences of bovine parvovirus and canine parvovirus. The identification results indicated that all 15 strains were clustered with bocavirus type 1 (Figure 3).

**Figure 4:**
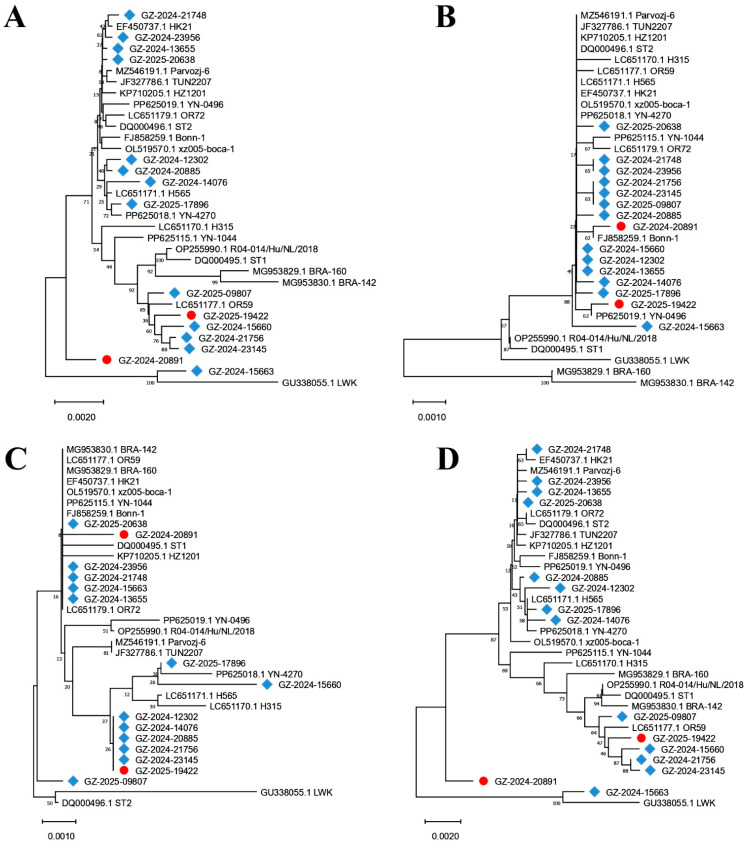
Phylogenetic analysis based on full length (**A**), NS1 (**B**), NP1 (**C**), and VP1/VP2 (**D**) gene. Mark ◆ denotes sequences obtained in our study. Mark ● denotes sequences obtained in this study for which it is speculated that gene recombination has occurred.

For further analysis, the nucleotide consistency between the 15 bocavirus sequences and each reference strain was 98.47~99.94%, and the nucleotide consistency between the 15 bocavirus sequences was 99.15~99.92%. The phylogenetic tree based on the full length of the bocavirus type 1 genome and three gene fragments (NS1, NP1, VP1/2) showed that the phylogenetic tree constructed with the whole genome was similar to that constructed with the gene VP1/2 (Figure 4).

### 3.4. Recombination Analysis

Among the 15 whole-genome sequences of bocavirus obtained in our study, the strains GZ-2024-20891 (GenBank accession No. PZ231451) and GZ-2025-19422 (GenBank accession No. PZ231458) were suspected to be recombined. A total of seven recombination detection algorithms from RDP4 were employed to screen, suggesting that the suspected recombination parents of strain GZ-2024-20891 were LWK and Bonn-1, which were supported by six algorithms in this potential recombination event. The suspected recombination parents of the strain GZ-2025-19422 were YN-0496 and BRA/TO-142, and the potential recombination events were supported by seven algorithms (Table 3).

**Figure 5:**
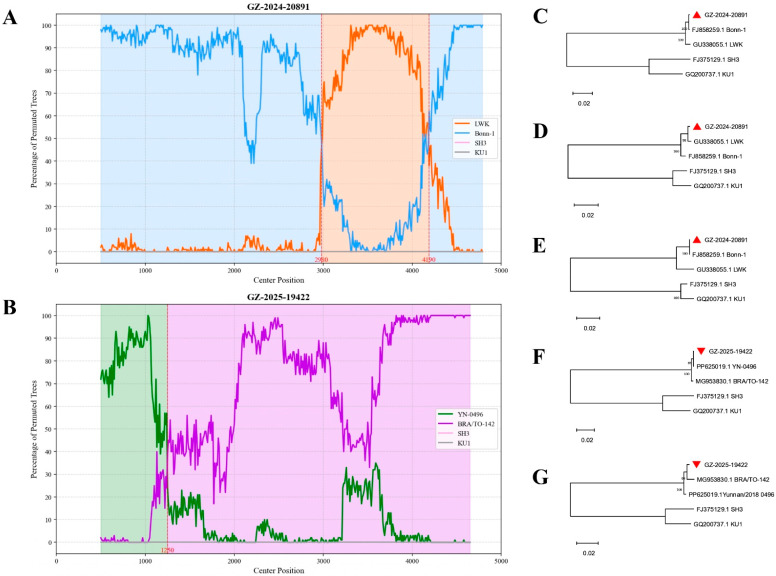
Simplot recombination detection results (**A**,**B**) and phylogenetic tree based on breakpoint partition sequences (**C**–**G**). Mark ▲ and ▼ denote suspected recombinant sequences GZ-2024-20891 and GZ-2025-19422, respectively. (**C**–**E**) The phylogenetic trees of sequence GZ-2024-20891 segmented into three parts: the segment before the first breakpoint, the segment between the two breakpoints, and the segment after the second breakpoint, respectively. (**F**,**G**) The phylogenetic trees of sequence GZ-2025-19422 segmented into two parts: the segment before the breakpoint and the segment after the breakpoint, respectively.

Further analysis of the two sequence groups was conducted using the BootScan algorithm in SimPlot software. Two distinct crossovers of the two putative parental signal lines were observed in strain GZ-2024-20891, with higher relatedness to strain Bonn-1 prior to 2980 nt. The first crossover of signal lines occurred at 2980 nt, suggesting a potential recombination breakpoint at this location. Subsequently, clustering relatedness to strain LWK predominated in the 2981–4190 nt region, followed by a second crossover of signal lines at 4190 nt, after which closer relatedness to strain Bonn-1 was restored (Figure 5A). The parental signal line of strain GZ-2025-19422 exhibits a recombination breakpoint at position 1250 nt. Prior to the breakpoint, the sequence shows closer phylogenetic relatedness to sequence YN-0496, whereas beyond the breakpoint, it demonstrates closer relatedness to sequence BRA/TO-142 (Figure 5B).

Taking the recombination breakpoints as boundaries, each segment of the sequence was partitioned and analyzed through separate phylogenetic tree construction. The results indicated that for strain GZ-2024-20891, the segments located outside the two recombination breakpoints (i.e., the first and third segments) clustered with Bonn-1, while the internal segment situated between the breakpoints clustered with LWK (Figure 5C,D). Meanwhile, for strain GZ-2025-19422, the segment preceding the breakpoint clustered with YN-0496, and the segment following the breakpoint clustered with BRA/TO-142 (Figure 5E–G). These findings are consistent with the results generated by the SimPlot BootScan program. Based on the results, we infer that the two sequences monitored in 2024 and 2025, respectively.

### 3.5. Comparison of Predicted Linear Epitope

Linear antigenic epitopes of the VP2 protein sequences from recombinant strain GZ-2024-20891 and its parental strains were predicted and compared. The results revealed in silico-predicted alterations in the antigenic characteristics of two B cell epitopes, two CD8+ T cell epitopes, and one CD4+ T cell epitope in the recombinant strain compared to the parental strains (Table 4). Using the same methodology, epitope predictions and comparisons between the recombinant strain and two domestically circulating strains (GZ-2024-15663 and YN-1044, both of which circulated in the same period) were conducted. The analysis identified seven epitopes in the recombinant strain that exhibited in silico-predicted changes relative to the circulating strains (Table 5).

### 3.6. Comparison of Predicted Conformational Epitope

**Figure 6:**
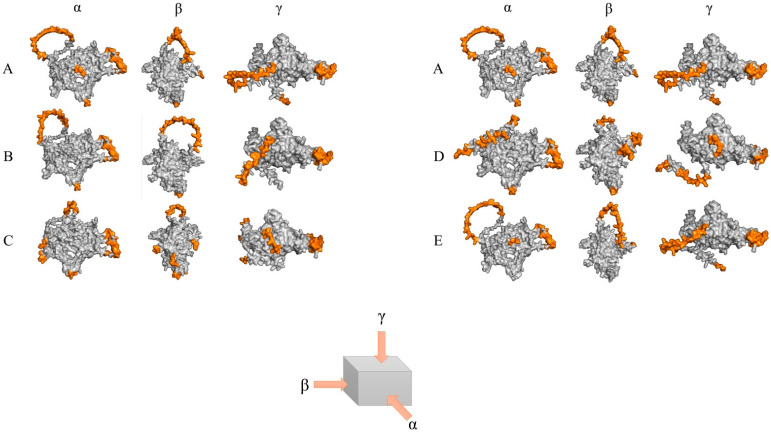
Three-dimensional visualization of predicted B cell conformational epitopes on the VP2 protein. A represents the predicted model of the VP2 protein of the recombinant strain GZ-2024-20891; B and C represent the models of the VP2 protein of the parental strain LWK and Bonn-1, respectively; and D and E represent the models of the VP2 protein of the circulating strain GZ-2024-15663 and YN-1044. α denotes the front view, β denotes the left-side view, and γ denotes the top view.

The three-dimensional structural models of the VP2 protein predicted for the recombinant strain, parental strains, and domestically circulating strains in the AlphaFold3 server all exhibited high prediction quality (pTM values of 0.86). Conformational epitopes predicted based on these structural models using ElliPro are illustrated in Figure 6. In comparison with parental and circulating strains, both the quantity and spatial localization of conformational epitopes in the recombinant strain were predicted to be altered (Figure 6). GZ-2024-20891 increased the epitopes of residues 1–5 compared with LWK; Compared with bonn-1, the epitopes of residues 30–48, 497–502 and 180–183 were missing. Compared with GZ-2024-15663, the epitopes of residues 24–29 and 19–23 were missing. Compared with YN-1044, the epitope of residues 27–29 was missing, and the epitope of residues 243–248 was increased (Table S1).

## 4. Discussion

Human bocavirus has become one of the most common viruses associated with acute respiratory infections in infants and young children. Recent studies have further confirmed its pathogenicity in respiratory infections and have progressively deepened the understanding of its clinical manifestations and pathogenic mechanisms. Given the significant disease burden imposed by bocavirus on the pediatric population and its prominent role in childhood respiratory infections, studies on bocavirus have been becoming increasingly important, especially in pathogen surveillance. This study, focusing on surveillance cases from sentinel hospitals in Guangzhou, described the epidemiological characteristics and co-infection patterns of bocavirus and revealed antigenic alterations following recombination events in bocavirus.

Respiratory specimens from surveillance cases were collected in this study between August 2023 and December 2025. In terms of temporal distribution, the epidemic peak of human bocavirus in Guangzhou predominantly occurred in autumn and the surrounding months, which is largely consistent with previous reports [28]. Notably, a secondary peak in bocavirus prevalence was observed shortly following the conclusion of the main autumn peak. We speculate this may be attributed to the extensive replication and widespread transmission of bocavirus during its primary peak period. With high prevalence, the likelihood of co-infection among bocavirus strains increases, thereby elevating the probability of recombination events [29,30], which may contribute to alterations in viral antigenicity. The immunity established within the population is specific to the circulating strain during the main epidemic peak. In this hypothetical context, it is possible that population immunity to potential recombinant strains is limited, but this remains to be tested.

In this study, bocavirus infection shows no significant gender differences and primarily affects infants and young children under 3 years old consistent with previous studies [28,31]. Co-infection analysis indicates that rhinovirus and parainfluenza virus are the two most common viruses associated with bocavirus co-infection, with both types of co-infection also occurring most frequently in individuals under 3 years of age. Analysis of the co-infection chi-square test of independence revealed that rhinovirus, parainfluenza virus, and influenza virus were more frequently co-detected with bocavirus. One possible explanation for this observed co-occurrence is the overlap in epidemic peaks and susceptible populations between the co-infection viruses. Previous studies indicate that influenza virus typically peaks during winter, partially overlapping with bocavirus’s peak season [32,33,34]. Though rhinovirus and parainfluenza virus circulate throughout the year, both viruses share pediatric populations as susceptible hosts [35,36,37,38], overlapping with the susceptible demographic of bocavirus. Whether bocavirus has a true biological preference for co-infection with these pathogens or whether co-infection enhances viral replication remains unknown and requires further experimental investigation.

Our study obtained 15 complete genome sequences of bocavirus type 1. Notably, phylogenetic analyses based on individual genes revealed divergent clustering patterns for strains GZ-2024-20891 and GZ-2025-19422 across different genes. The GZ-2024-20891 sequence clustered with the German strain Bonn-1 in the phylogenetic tree based on the NS1 gene but remained relatively independent from Bonn-1 in trees based on the NP1 and VP1/2 genes [39]. The GZ-2025-19422 sequence clustered with the Chinese Yunnan strain YN-0496 based on the NS1 gene but showed greater divergence based on the NP1 and VP1/2 genes, which led to speculation around the possibility that recombination events may have occurred in these two strains.

Recombination analysis conducted using the RDP4 program revealed evidence suggestive of recombination events in both of the obtained sequences. For sequence GZ-2024-20891, the major parental strain was identified as a Chinese Guangzhou isolate LWK, while the minor parental strain was a German isolate Bonn-1. For sequence GZ-2025-19422, the major parental strain was a Chinese Yunnan isolate YN-0496, and the minor parental strain was a Brazilian isolate BRA/TO-142. Analysis using the BootScan algorithm in SimPlot software confirmed the aforementioned putative recombination events. The signal lines of recombinant parent sequence GZ-2024-20891 exhibits distinct crossovers at positions 2980 nt and 4190 nt, indicating the presence of recombination breakpoints. The signal lines of sequence GZ-2025-19422 shows a recombination breakpoint at position 1250 nt. To validate the reliability of the detected recombination events, phylogenetic trees were constructed separately for each sequence segment divided based on the identified recombination breakpoints. The results demonstrated that both sequences exhibited a distinct “tree-jumping” phenomenon, where the phylogenetic positions of different sequence segments underwent significant shifts, revealing inconsistencies in phylogenetic clustering. This provides further supportive evidence for the occurrence of recombination events.

Current studies have indicated that the VP2 protein serves as an antigen for bocavirus type 1 [40,41,42]. Notably, the strain GZ-2024-20891, located at the 4190 nt recombination breakpoint, situates within the coding region of the bocavirus VP2 protein [43]. Consequently, the antigenicity of this bocavirus strain may be altered as a result of this recombination event. Potential linear epitopes of the recombinant strain VP2 protein were compared with those of the parental strains, revealing that five linear epitopes showed predicted differences compared to the parental strain. Among these, four epitopes were similar to parental strain Bonn-1, and one epitope was similar to LWK. Mapping of the corresponding amino acid residue positions to the genome demonstrated that the four epitopes similar to Bonn-1 were all located downstream of recombination breakpoint 4190 nt, while the single epitope similar to LWK spanned recombination breakpoint 4190 nt, consistent with the recombination pattern displayed by SimPlot. Further comparative analysis of conformational epitopes between the recombinant and parental strains indicated significant alterations in both the quantity and spatial localization of conformational epitopes. Based on above evidence, we suggest that the recombination event may contribute to an alteration in the antigenicity of the VP2 protein in the recombinant strain.

Furthermore, comparing the linear epitopes of the VP2 protein from the recombinant strain with the sequence of the domestically circulating strains GZ-2024-15663 and YN-1044 revealed predicted epitope differences in antigenic characteristics at seven potential linear epitopes. Additional comparative analysis of conformation epitopes between the recombinant and circulating strains similarly indicated significant spatial alterations using in silico methods. Based on these findings, it is suggested that the antigenicity of the VP2 protein in the recombinant strain differs from that of the domestically circulating strain.

In consideration of the epidemiological characteristics of bocavirus, the antigenic alteration associated with this recombination event may be correlated with the secondary epidemic peak of bocavirus. We hypothesize that recombination-associated antigenic changes may contribute to immune escape against the recombinant strain, rendering immune protection ineffective in a subset of the population, thereby possibly playing a role in the secondary peak. Although this study did not directly demonstrate the occurrence of immune evasion through experimental validation, based on the consistent evidence from the viral epidemiological characteristics, recombination events, and antigenic alterations, we propose that vigilance should be maintained regarding recombinant strains of human bocavirus that emerge during epidemic peaks. If surveillance identifies recombinant strains with altered antigenic characteristics, such findings should serve as an early warning signal to raise attention.

There are a few limitations in this study. The analysis of the antigenic characteristics of the VP2 protein was based entirely on in silico epitope prediction and has not been experimentally validated. It remains to be confirmed whether changes in the binding capacity of viral particles as antigens to antibodies or immune cells have occurred. Therefore, the findings only allow for speculation regarding whether antigenic alterations have taken place in the recombinant strain, without directly demonstrating enhancement or attenuation of the antigenicity of the VP2 protein. Furthermore, our observation period spans from August 2023 to December 2025. Because the window does not include complete calendar years, the identified seasonal peaks should be interpreted with caution.

To build upon the present findings, future research should focus on three main directions. First, functional validation of the predicted antigenic alterations is required to determine whether the identified recombination events indeed alter viral antigenicity. Second, temporal analyses and population immunity studies are needed to assess whether the secondary epidemic peak can be attributed to recombination and antigenic changes. Third, enhanced genomic surveillance of bocavirus should be implemented in routine public health practice to monitor the emergence and spread of recombinant strains and potential antigenic variants in real time.

## 5. Conclusions

In summary, this study describes the epidemiological characteristics and co-infection patterns of bocavirus in Guangzhou, revealing that bocavirus is highly prevalent among infants and young children under 3 years of age, with a secondary peak following the main epidemic peaks around autumn. Co-infections with rhinovirus and parainfluenza virus are frequently observed. Recombinant events and in silico differences in epitopes were detected, and both findings warrant expanded genomic surveillance and functional validation.

## Figures and Tables

**Table 1:** Positive rate of bocavirus in the population.

		Number of Positive Cases	Total	Positive Rate (%)	*χ* ^2^	*p*	OR (95% CI)
Age					64.371	0.000	
	0–2	41	1030	3.98	-	-	-
	3–5	21	1278	1.64	11.920	0.001	0.403 (0.237–0.686)
	6–17	12	1563	0.77	32.008	<0.001	0.187 (0.098–0.357)
	18–64	3	1079	0.28	35.364	<0.001	0.067 (0.021–0.218)
	≥65	1	366	0.27	12.719	<0.001	0.066 (0.009–0.482)
	Total	78	5316	1.47			

Note: For all *χ*^2^ tests and odds ratios, the reference category for age was the 0–2 year group.

**Table 2:** Number of cases co-infected with bocavirus.

		BoV (+)	BoV (−)	Total	Co-Infection Rate	*χ* ^2^	*p*	OR (95% CI)
IV					1.28% (1/78)	13.159	0.000	0.065 (0.009–0.470)
	IV (+)	1	869	870				
	IV (−)	77	4369	4446				
	Total	78	5238	5316				
RSV					2.56% (2/78)	0.830 ^a^	0.362	0.444 (0.109–1.817)
	RSV (+)	2	293	295				
	RSV (−)	76	4945	5021				
	Total	78	5238	5316				
PIV					11.54% (9/78)	11.827 ^a^	0.001	3.563 (1.752–7.246)
	PIV (+)	9	185	194				
	PIV (−)	69	5053	5122				
	Total	78	5238	5316				
RV					23.08% (18/78)	17.129	0.000	2.940 (1.722–5.020)
	RV (+)	18	485	503				
	RV (−)	60	4753	4813				
	Total	78	5238	5316				
hMPV					5.13% (4/78)	0.011 ^a^	0.917	1.221 (0.443–3.370)
	hMPV (+)	4	222	226				
	hMPV (−)	74	5016	5090				
	Total	78	5238	5316				
AdV					2.56% (2/78)	0.213 ^a^	0.645	0.589 (0.144–2.414)
	AdV (+)	2	224	226				
	AdV (−)	76	5014	5090				
	Total	78	5238	5316				
SARS-CoV-2					0.00% (0/78)	2.731 ^a^	0.098	0.264 (0.037–1.908) ^b^
	SARS-CoV-2 (+)	0	242	242				
	SARS-CoV-2 (−)	78	4996	5074				
	Total	78	5238	5316				

Note: ^a^ denotes the corrected chi-square test statistic; ^b^ denotes a correction of 0.5 added to each cell of the 2 × 2 table due to the presence of zero cells.

**Table 3:** RDP4 recombination detection results.

Recombinant Strain	Major Parent	Minor Parent	Recombination Algorithm Detection Results (Result/*p* Value)						
			RDP	GENECONV	BootScan	MaxChi	Chimera	SiScan	3Seq
GZ-2024-20891	LWK	Bonn-1	-/-	+/4.009 × 10^−5^	+/4.716 × 10^−6^	+/3.204 × 10^−4^	+/2.010 × 10^−4^	+/4.922 × 10^−3^	+/3.596 × 10^−6^
GZ-2025-19422	YN-0496	BRA/TO-142	+/2.606 × 10^−4^	+/4.099 × 10^−3^	+/9.597 × 10^−3^	+/4.837 × 10^−4^	+/2.449 × 10^−3^	+/5.484 × 10^−11^	+/7.091 × 10^−5^

**Table 4:** Comparison of linear epitopes between recombinant and parental strains.

Epitope Type	Epitope	aa (Start–End)	nt (Start–End)	GZ-2024-20891 (Value/Result)	LWK (Value/Result)	Bonn-1 (Value/Result)
B cell	A/A’	324–339	4296–4341	0.8841/PA	0.7929/PNA	0.8841/PA
B cell	B/B’	317–336	4275–4332	0.9119/PA	0.8197/PNA	0.9119/PA
CD8+ T cell	C/C’	450–458	4674–4698	1.0207/PA	0.6714/PNA	1.0207/PA
CD8+ T cell	D/D’	320–328	4284–4308	0.9138/PA	0.7040/PNA	0.9138/PA
CD4+ T cell	E/E’	286–300	4182–4224	0.9029/PA	0.9029/PA	0.8326/PNA

Note: A: TGRIQPYSKPTSWMTG; A’: TSRIQPYSKPTSWMTG; B: NGSTAASTGRIQPYSKPTSW; B’: NGSTAASTSRIQPYSKPTSW; C: FPITRENPI; C’: YPITRENPI; D: TAASTGRIQ; D’: TAASTSRIQ; E: CEWVNNERAYIPPGL; E’: CEWINNERAYIPPGL; PA: Probable Antigen; PNA: Probable Non-Antigen.

**Table 5:** Comparison of linear epitopes between recombinant and domestically circulating strain.

Epitope Type	Epitope	aa (Start–End)	nt (Start–End)	GZ-2024-20891 (Value/Result)	GZ-2024-15663 (Value/Result)	YN-1044 (Value/Result)
B cell	F/F’	324–339	4296–4341	0.8841/PA	0.7929/PNA	-/-
B cell	G/G’	317–336	4275–4332	0.9119/PA	0.8197/PNA	-/-
CD8+ T cell	H/H’	320–328	4284–4308	0.9138/PA	0.7040/PNA	-/-
CD8+ T cell	I/I’	450–458	4674–4698	1.0207/PA	0.6741/PNA	-/-
B cell	J/J’	180–187	3864–3885	0.8109/PNA	-/-	1.1187/PA
CD8+ T cell	K/K’	178–186	3858–3882	0.7003/PNA	-/-	0.8750/PA
CD4+T cell	L/L’	181–195	3867–3909	0.8473/PNA	-/-	0.9153/PA

Note: F: TGRIQPYSKPTSWMTG; F’: TSRIQPYSKPTSWMTG; G: NGSTAASTGRIQPYSKPTSW; G’: NGSTAASTSRIQPYSKPTSW; H: TAASTGRIQ; H’: TAASTSRIQ; I: FPITRENPI; I’: YPITRENPI; J: NGADTTYN; J’: NGTDTTYN; K: LSNGADTTY; K’: LSNGTDTTY; L: GADTTYNNDLTAGVH; L’: GTDTTYNNDLTAGVH; PA: Probable Antigen; PNA: Probable Non-Antigen.

## Data Availability

The original data presented in the study are openly available in GenBank repository under accession No. PZ231445-PZ231459.

## Supplementary Materials

The following supporting information can be downloaded at: https://www.mdpi.com/article/10.3390/v18060686/s1, Table S1: Result of predicted conformational epitope.

## Abbreviations

The following abbreviations are used in this manuscript:

HBoV	Human Bocavirus
IV	Influenza Virus
RSV	Respiratory Syncytial Virus
PIV	Parainfluenza Virus
RV	Rhinovirus
hMPV	Human Metapneumovirus
AdV	Adenovirus
SARS-CoV-2	Severe Acute Respiratory Syndrome Coronavirus 2
ILI	Influenza-like Illness
SARI	Severe Acute Respiratory Infection
