# Supplementary material for: Analysis of Epidemiological and Molecular Characteristics of Bocavirus in Guangzhou
Source: Viruses. 2026 Jun 20;18(6):686. doi: 10.3390/v18060686 (PMC13307671; doi:10.3390/v18060686)
Supplement: Supplementary file 1 [file viruses-18-00686-s001.zip › viruses-4376575-supplementary.pdf]

Supplementary Table S1. Result of predicted conformational epitope

Srain	Epitope number	Epitope position	Residues	Score
GZ-2024-20891	1	30-48	KSEPKPGTSKMSDTDIQDQ	0.965
	2	49-55	QPDTVDA	0.933
	3	303-308, 310-311, 313-314, 316-327, 329, 430	NPKVPT-RV-YI-QNGSTAASTGRI-P---Q	0.923
	4	1-5	VAPAL	0.922
	5	243-248	GNAAGG	0.918
LWK	1	30-48	KSEPKPGTSKMSDTDIQDQ	0.973
	2	49-55	QPDTVDA	0.940
	3	303-308, 310-314, 316-330, 429-431	NPKVPT-RVQYI-QNGSTAASTSRIQPY--NQT	0.916
	4	243-248	GNAAGG	0.909
Bonn-1	1	49-60	QPDTVDAPQNTS	0.955
	2	303-308, 310-314, 316-330, 332, 429-432	NPKVPT-RVQYI-QNGSTAASTGRIQPY-K---NQTT	0.939
	3	497-502	PTASNA	0.920
	4	242-250, 366, 368	DGNAAGGNA--G-H	0.918
	5	180-183	NGAD	0.913
	6	1-4	VAPA	0.900
GZ-2024-15663	1	24-29	GAKKTK	0.970
	2	1-7, 9-10	VAPALGN-ER	0.968
	3	243-249	GNAAGGN	0.928
	4	306-308, 310-327, 329, 429-431	VPT-RVQYIRQNGSTAASTSRI-P---NQT	0.924
	5	19-23	ANSNK	0.923
	6	30-32	KSE	0.914
	7	50-57	PDTVDAPQ	0.914
YN-1044	1	30-48	KSEPKPGTSKMSDTDIQDQ	0.980
	2	49-55	QPDTVDA	0.947
	3	303-308, 310-314, 316-331, 430	NPKVPT-RVQYI-QNGSTAASTGRIQPYS--Q	0.915
	4	27-29	KTK	0.911
	5	1-4	VAPA	0.907
